# Deformation twinning induced decomposition of lamellar LPSO structure and its re-precipitation in an Mg-Zn-Y alloy

**DOI:** 10.1038/srep30096

**Published:** 2016-07-20

**Authors:** X. H. Shao, S. J. Zheng, D. Chen, Q. Q. Jin, Z. Z. Peng, X. L. Ma

**Affiliations:** 1 Shenyang National Laboratory for Materials Science, Institute of Metal Research, Chinese Academy of Sciences, 72 Wenhua Road, 110016 Shenyang, China

## Abstract

The high hardness or yield strength of an alloy is known to benefit from the presence of small-scale precipitation, whose hardening effect is extensively applied in various engineering materials. Stability of the precipitates is of critical importance in maintaining the high performance of a material under mechanical loading. The long period stacking ordered (LPSO) structures play an important role in tuning the mechanical properties of an Mg-alloy. Here, we report deformation twinning induces decomposition of lamellar LPSO structures and their re-precipitation in an Mg-Zn-Y alloy. Using atomic resolution scanning transmission electron microscopy (STEM), we directly illustrate that the misfit dislocations at the interface between the lamellar LPSO structure and the deformation twin is corresponding to the decomposition and re-precipitation of LPSO structure, owing to dislocation effects on redistribution of Zn/Y atoms. This finding demonstrates that deformation twinning could destabilize complex precipitates. An occurrence of decomposition and re-precipitation, leading to a variant spatial distribution of the precipitates under plastic loading, may significantly affect the precipitation strengthening.

Precipitation hardening is widely applied in engineering materials, based on the fact that the small-scale precipitates (usually with complex crystal structure) play a critical role in resisting dislocation motion. Generally, small-scale precipitates display coherent or semi-coherent interfaces with the matrix. The quantity, morphology, and distribution of these small precipitates are of critical importance in tuning mechanical properties of the materials.

Understanding the mechanisms of evolution of the precipitates during plastic deformation is thus one of the central issues for advanced materials design. Many investigations on steel and Al alloys confirm that the precipitations can be decomposed during mechanical loading^1^,^2^,^3^,^4^,^5^,^6^, leading to a detrimental consequence of mechanical performance. The decomposition of precipitates is generally attributed to the interaction between the precipitations and defects at the interface^1^,^2^,^3^,^4^,^5^,^7^ or within the precipitates^6^,^8^. However, in hexagonal-close-packed (HCP) metals and alloys, the influences of defects on precipitates are less understood. Twinning is one of the dominant deformation mechanisms in HCP lattice owing to their very limited independent slip systems^9^. For the frequently observed 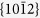 deformation twin, the twin boundaries (TBs) are often serrated rather than fully coherent. These serrated TBs consist of segment of coherent TBs and the prismatic/basal (PB) or basal/prismatic (BP) interfaces resulted from mechanical and/or thermal loadings^10^,^11^,^12^,^13^,^14^,^15^. The deformation-induced PBs/BPs interface was experimentally observed in pure magnesium^10^, pure cobalt^11^,^12^ and AZ31^13^. Such an interface features with an array of misfit dislocations to relieve the lattice mismatch between basal and prismatic planes. However, detailed information on the interaction between misfit dislocations and the precipitates in the HCP alloys is still unknown. Thus, the objective of this study is to clarify the distribution of misfit dislocations in the PBs/BPs interface and their effects on the stability of the precipitations in HCP alloys.

In this work, we report deformation twinning induced decomposition and re-precipitation of lamellar LPSO structures in an Mg-Zn-Y alloy, where the re-precipitation of lamellar LPSO structures in the twin especially represent the stacking faults (SFs) enriched with Zn/Y. The array of misfit dislocations along the lamellar LPSO-twin boundary (LPSO-TB) could enhance diffusion of the solute atoms along the basal planes. The present results demonstrate the influence of dislocations associated with the PB/BP interface on the re-ordering of secondary phase in Mg alloys. This work also indicates that the interaction between dislocations and solute atoms should be taken into account in understanding a material’s performance under plastic deformation.

## Results

### 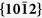 Deformation twinning

**Figure 1:**
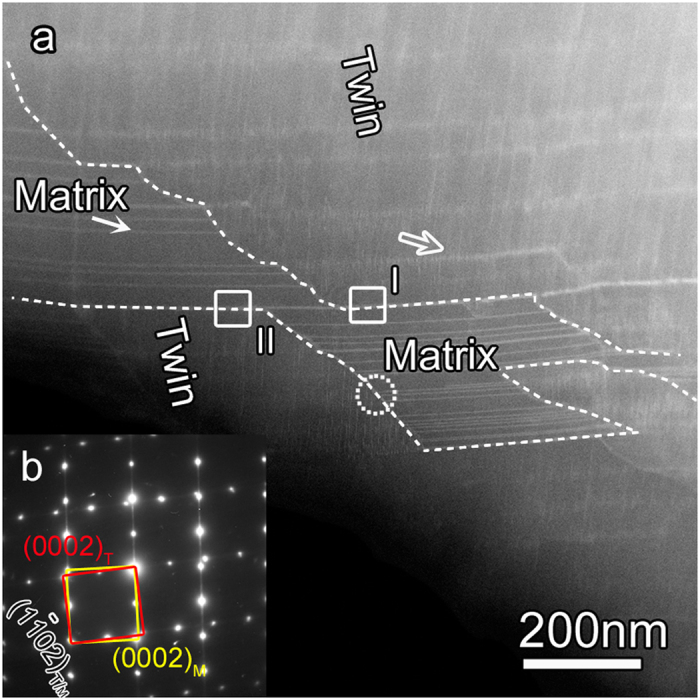
(**a**) A low magnified STEM image showing the microstructure of a deformation twin in the Mg_97_Zn_1_Y_2_ alloy after compression at 573 K. (**b**) A corresponding SAED pattern taken along [11
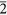
0] from the circled area, demonstrating it’s a 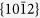 deformation twin.

The Mg_97_Zn_1_Y_2_ (at.%) alloy experienced a hot compression at 573 K and at a constant stain rate of 10^−1^s^−1^. The compression strain-stress curve is shown in Supplementary Fig. S1. A few deformation kinks formed in the LPSO structures in the deformed sample, as marked by arrows in Supplementary Fig. S2, similar to the phenomenon in the previous work^16^. In this work, we focused on the evolution of lamellar LPSO /SFs enriched with Zn/Y in the matrix of Mg alloys during twinning. Figure 1a shows a low magnification STEM image of a region containing a 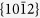 deformation twin in the deformed Mg-Zn-Y alloy, where the TB was delineated by the white dashed line. The diffraction pattern of the deformation twin, circled in Fig. 1a, is indexed in Fig. 1b. The matrix is surrounded by the 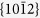 deformation twin. Profuse lamellar LPSO structures are parallel to the basal planes both in matrix and in deformation twin, which feature long bright lines and short bright lines and marked with the solid and open arrows, respectively. The TBs consist of segments of lamellar LPSO and rugged TBs which are almost parallel to the 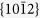 plane. Interestingly, the TB located at the LPSO structures seems like the BPs/PBs observed in pure Mg^10^, Co^11^,^12^, and AZ31^13^, giving rise to an orientation relationship identical to that of the 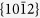 deformation twin. The formation of BPs/PBs is geometrically and energetically preferred in Mg and Co evidenced via simulation^14^. Also in some Mg alloys containing secondary phases, similar configuration has been observed during twinning, such as the TB between matrix and *β* phase in AZ91^17^,^18^ and TB bypassing the low density of long period stacking ordered (LPSO) phase in Mg-Zn-Y alloy^19^. Therefore, the LPSO-TB interface observed here is representative in HCP metallic alloys, and an in-depth study of its formation mechanisms will be of great importance in engineering materials.

### LPSO-twin boundary interfaces

**Figure 2:**
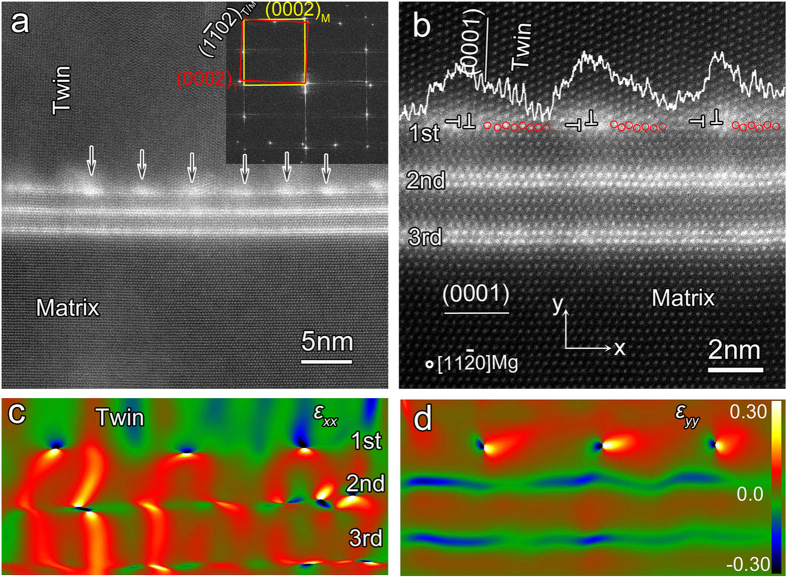
(**a**) A high magnified STEM image corresponding to the framed area I in Fig. 1(a), and the corresponding Fast Fourier Transform (FFT), inset in Fig. 2(a), demonstrating the upper and lower part exhibiting the 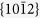 twinning orientation relationship. (**b**) High-resolution STEM image of rugged twin boundary formed by segments of LPSOs, showing periodic dislocations along the interface. The GPA strain map (**c**) parallel with (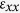) and (**d**) perpendicular to (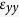) the LPSO-twin interface in (**b**), exhibiting misfit dislocation cores with sharp strain gradient.

The LPSO-TB interface contains an array of misfit dislocations, deviating from the original stacking sequence and stoichiometric ratio of lamellar LPSO structures. The high magnification STEM observations reveal that the interface between lamellar LPSO structures and twins, marked by rectangles *I* in Fig. 1a, in fact exhibit periodic bright contrast, as shown by the arrows in Fig. 2a. The Fast Fourier Transformation (FFT) acquired across the boundary shows a rotation of 86° between the matrix and the deformed region, exhibiting a 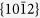 twin relationship, as illustrated by the inset of Fig. 2a. This local configuration is similar to the twinning-like lattice reorientation happened in single-crystal pure magnesium^10^. The different twin angles between the twinning like BP/PBs (90°)^10^ and LPSO-TB (86°) should be attributed to shuffling mechanism for the former and the shuffling and twinning dislocation for the latter, respectively. The high-resolution STEM observations show that the dislocation spacing at the LPSO-TB interface is approximately 4.0 nm (Fig. 2b). On the one hand, the unique LPSO-TB could be considered as the {0001}<1
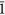
00> BPs with respect to the basal plane of twin^10^,^11^,^14^. On the other hand, this kind of LPSO-TB interface could also be considered as a {0001}<1
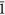
00> BPs with respect to the basal plane of matrix. The one-dimensional lattice fringe images obtained using a Fast Fourier Transformation (FFT) filtering process showing two arrays of misfit dislocations at the LPSO-TB interface, as illustrated in Supplementary Fig. S3. The semi-coherent LPSO-TB interface contains arrays of misfit dislocations owing to the mismatch of lattices 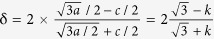 = 6.5% along the LPSO-TB, *k = c/a *= 1.623 for Mg. The average spacing is calculated about 4.0 nm, which is confirmed by the periodic dislocation distribution shown in Fig. 2b. The array of periodic dislocations parallel to the interface (Supplementary Fig. S3b) lead to a small angle, approximately 3.7°, fitting well with the twin orientation relationship and our observations (Fig. 2a). The 1^st^, 2^nd^, and 3^rd^ LPSO indicate the layers enriched with Zn/Y atoms in LPSO structures based on the departing distance from the layers to the LPSO-TB interface, respectively, as denoted in Fig. 2b. The short segment 1^st^ LPSO between the dislocations deviates from the AB′C′A stacking sequence, and locally has been reoriented into twinning, indicated by red circles in Fig. 2b. Furthermore, the dislocations exhibit higher contrast probably because they trap some Zn/Y atoms, which may result in a lower energy for the total system^20^. This is confirmed by the line intensity profile of the 1^st^ LPSO structures, inset in Fig. 2b. The fluctuant line intensity profile also suggests the composition deviates from the stoichiometric ratio in the 1^st^ lamellar LPSO, which should be closely related with the dislocations or defects in the LPSO-TB interface during deformation. To clearly display the dislocation cores in the interface of LPSO structure and twin in Fig. 2b, the strain maps parallel to ([1
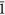
00] direction for matrix and [0001] direction for twin, X direction) and perpendicular to ([0001] direction for matrix and [1
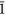
00] direction for twin, Y direction) the interface obtained by geometric phase analysis (GPA)^21^,^22^ are provided in Fig. 2c,d. The strain distribution and extra half planes at the LPSO-TB interface (Supplementary Fig. S3), illustrating the distribution of compression and tensile strain of the periodic dislocations. Meanwhile, the strain distributing on and between the 1^st^, 2^nd^, 3^rd^ position is proposed to be originated from the different microstructure of AB′C′A building block (FCC) and Mg interlayers (HCP). The high magnification and atomic resolution of STEM observations of the region marked by rectangle *II* in Fig. 1a, demonstrate the region *II* has the similar microstructural features with that of region *I* (not shown here). The 2^nd^ and 3^rd^ lamellar LPSO, even the Mg planes sandwiched in these lamellar LPSO, remain the original stacking sequence and chemical composition. This may indicate that the outermost layer of LPSO could act as mechanical hard shield protecting LPSO during shearing.

### Stacking faults re-precipitation in deformation twin

**Figure 3:**
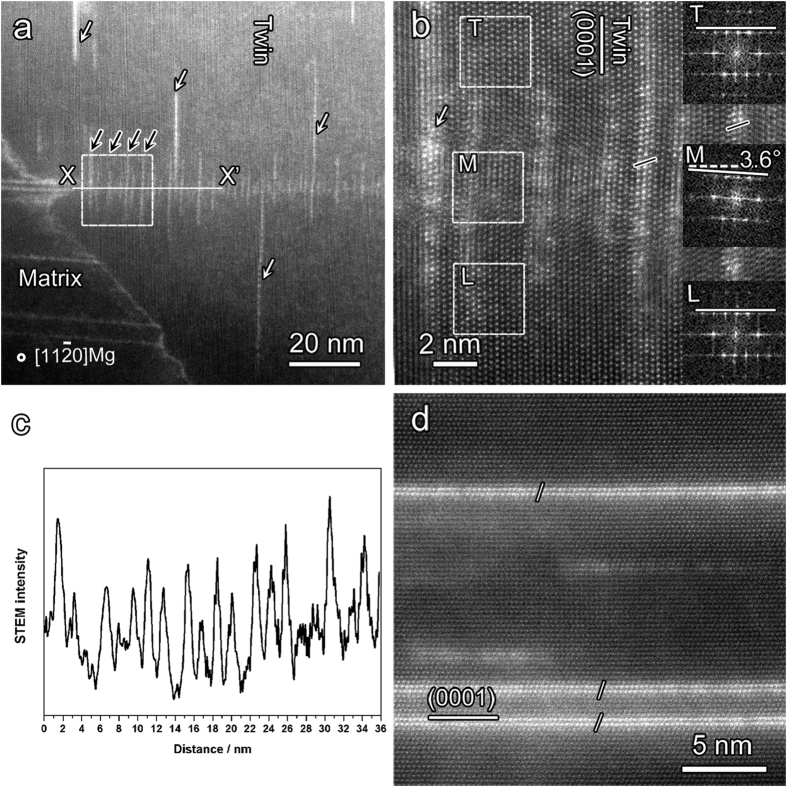
(**a**) A typical microstructure of precipitated SF (T-SFs) within deformation twin, just at the localization of original SFs. (**b**) High-resolution STEM images for the framed region in Fig. 3(a), indicating there is some curvature and lattice distortion existing in the T-SFs. The inset Fast Flourier Transformation (FFT) patterns of areas denoted by T, M, and L clearly demonstrate the local distortion of basal planes in T-SFs. (**c**) A line profile across II T-SFs along X-X’ in Fig. 3a, showing they depart from each other about 1.6~2.0 nm. (**d**) The SFs in the as-cast Mg-Zn-Y alloys, obviously different from the T-SFs considering the length scale and lattice distortion.

Figure 3a shows a low magnification STEM image recorded from the rugged 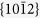 TB. In the twin, planar defects are present along the basal plane in deformation twin but they are almost perpendicular to the original LPSO phase, as shown by the long bright lines in the matrix. Several planar defects longer than 30 nm, denoted by the white arrows in Fig. 3a, randomly distributed in the twin. These are one kind of SFs, which are proposed to result from the dissociation of “**a**” or “**a + c**” dislocations in an Mg-Zn-Y alloy^23^. Figure 3b is a high-resolution STEM image of the interested region framed by a square in Fig. 3a. This reveals that the planar defects with AB′C′A blocks are enriched with Zn/Y (marked by short solid lines), and it also implies that SFs precipitated in the twin (T-SFs). The Fast Fourier Transformation (FFT) of the areas marked with T, M and L are inserted in Fig. 3b, which suggests that the basal plane of the T-SFs locally rotated about 3.6° relative to that of the upper and lower part in the twin. It should be noted that there is a dislocation at the upper left of “M”. The strain of this dislocation may contribute to the lattice distortion of 3.6°. In fact, similar lattice distortion is usually detected along the strings of T-SFs in the twin (e.g., original LPSO-TB interfaces) associated with residual dislocations. Thus the lattice rotation observed here should be originated from the residual dislocations during the interaction between LPSO and deformation twin. Here, the T-SFs in the twin are classified into two types according to the distribution of T-SFs: I T-SFs (randomly in the twin) and II T-SFs (along original LPSO-TB interfaces). Figure 3c is a line profile of the HAADF signal of II T-SFs along X-X’ in Fig. 3a, demonstrating the spacing of II T-SFs ranges from 1.6 to 2.0 nm. This space ties in with the separation distance of the FCC building blocks of 14H, 18R and 24R LPSO phases in Mg-Zn-Y alloy along *c*-axis (1.82 nm, 1.56 nm, and 2.04 nm, respectively), which implies that the arrays of II T-SFs should be the embryo of LPSO phase precipitated in twin. Meanwhile, a few II T-SFs with about 20 nm in length (marked by black arrows in Fig. 3a) may be particularly relevant to dislocations distributed at the LPSO-TB interface. The II T-SFs that nucleated at the misfit dislocations should be longer than the others due to the fast diffusion path, since solute atoms tend to segregate near the dislocations along LPSO-TB interface during deformation (Fig. 2). The dynamic nucleation of precipitations closely related to dislocations has been reported in various alloys, such as in Al-Cu-Mg^24^,^25^ and Al-Zn-Mg alloy^26^, which can attribute to the high creep resistance of the alloy. The precipitation of solute atoms and the dissociation of dislocations hence are proposed to rationalize the shorter (II T-SFs) and longer SFs (I T-SFs) in deformation twin, respectively. In addition, there’s obvious difference between the above observed T-SFs and original SFs at as-cast state, as far as the length scale and the lattice strain state are taken into account. As shown in Fig. 3d, the original SFs with AB′C′A stacking sequence formed during solidification are always about dozens or hundreds of nanometers in length and without obvious lattice strain. Hence, the T-SFs or the embryo of LPSO are the product of the evolution of lamellar LPSO phase during twinning. It is worthwhile to note that not every lamellar LPSO structure in the matrix transform into T-SFs in the twin. Instead, only the LPSO structure marked with the white arrow transforms into T-SFs in the twin, as shown in Fig. 3a. This suggests that the formation of T-SFs should be dependent on the local Zn/Y concentration and diffusion rate related to the local strain.

### Rugged 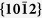 twin boundary consisting of BP and PB interfaces

**Figure 4:**
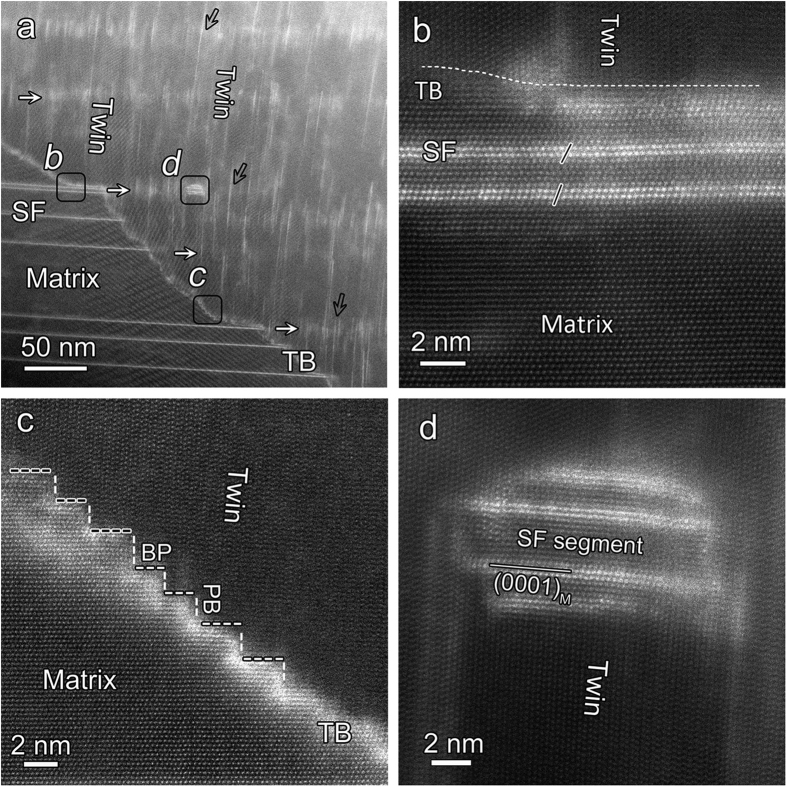
(**a**) A typical microstructure of deformation twin boundaries formed when twin encountering the multiple SFs. (**b**) An BP interface between the SF and twin, marked by *b* in Fig. 4a, where no misfit dislocation exists along this interface. (**c**) An alternate BP/PB interface forming twin boundary, framed by *c* in Fig. 4a. Black and white dotted lines represent BP and PB interfaces, respectively. (**d**) A short origional SFs about 8 nm in length remain within the deformation twin.

Figure 4a shows the rugged twin boundary almost along 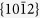 planes comprising profuse BPs and PBs interfaces at an atomic level. The array of a number of vertical T-SF in the twin is approximately parallel to the basal plane of original LPSO structure, as shown by arrows in Fig. 4a. The angle between basal plane of original LPSO structure and that of T-SFs is approximately equal to 92°, a little deviation from the ideal twin orientation relation (86.3°). This may be ascribed to the segmented composition of BP and PB interfaces along the 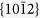 twin boundary^27^. Interestingly, it can be observed that the T-SFs are nearly vertical to the basal plane of original LPSO structures of the matrix (solid arrows), e.g., almost parallel to each other. By contrast, it’s more obvious that the basal planes of T-SFs in the twin are not parallel to each other farther from the twin boundary (open arrows). The precipitated LPSO/T-SFs in the twin is intimately related to the local basal plane. Such rotation of basal planes in the twin is proposed to be due to the subsequent plastic deformation in the twin region after twinning process. A little bit longer BP interfaces result from the intersection of the deformation twin and the lamellar LPSO structure, as shown in Fig. 4b, which could hinder the propagation of deformation twin to some extent^28^. The BPs and PBs are as thin as 2 nm. They are perpendicular to each other which give rise to a stepped boundary between the twinned and untwined regions, as demonstrated in Fig. 4c. These types of interfaces with bright contrast imply that they are enriched with solute atoms, different from that in pure metals^10^,^12^. Interestingly, a small segment of original SF, about 10 nm in length, remains in the deformation twin, as shown in Fig. 4d. Misfit dislocations are not observed along the outer layer of this remain, implying that during twinning fast path is not available for the solute atoms to transmit from matrix into the deformation twin. The Mg layers sandwiched in the SFs are stable and remain the matrix orientation, which is another proof that the layers enriched with Zn/Y layers could protect the inner structures during deformation.

## Discussion

### Mechanism for redistribution of Zn/Y atoms during twinning

**Figure 5:**
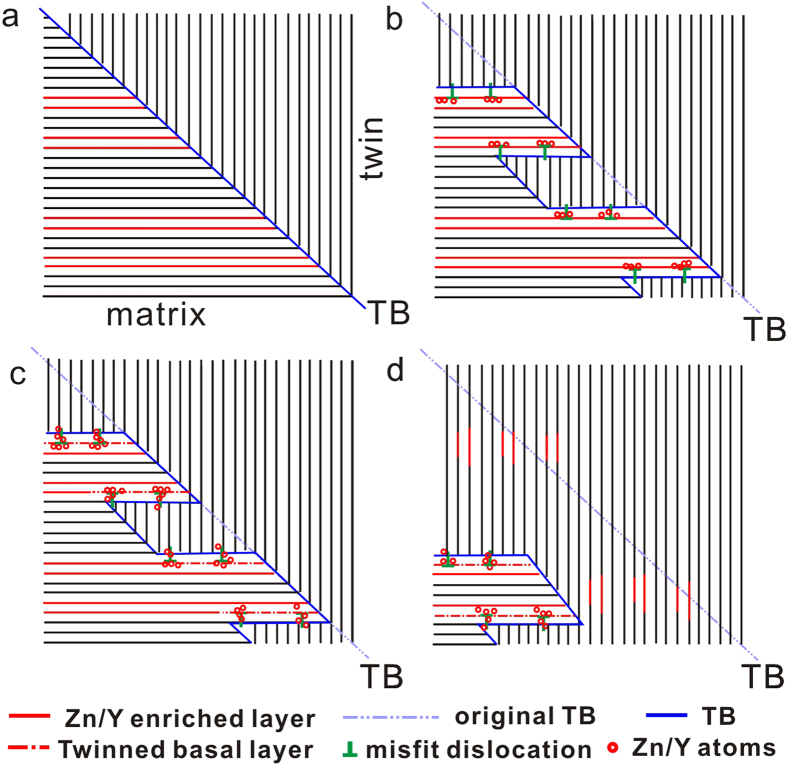
The illustration of microstructure evolution of the lamellar LPSO phase/SF in Mg-Zn-Y alloy during hot deformation. For simplicity, only one type of dislocations are denoted along LPSO-TB interface. Note that the convex border and terraces of the TB illustrated here at the intersection of the twin and LPSO structures, suggesting that the LPSO structure could pin the migration of twin boundaries.

On the basis of the above STEM observations, we propose the following mechanism for the decomposition and re-precipitation of lamellar LPSO phase in an Mg-Zn-Y alloy during twinning, as schematically illustrated in Fig. 5. It is well known that the lamellar LPSO structures in Mg-Zn-Y alloys are parallel to the basal plane of the Mg matrix (Fig. 5a). At the early stage of twinning growth, the LPSO-TB interface is associated with a periodic array of misfit dislocations. The Zn/Y atoms are attracted to the dislocations distributed at the interface via diffusing along basal planes in the matrix, resulting in a local disorder of the 1^st^ SF (Fig. 5b). Subsequently, the supersaturated Zn/Y atoms segregated at dislocations diffuse into the basal planes of the twin, illustrated in Fig. 5c. In the Mg lattice, diffusion rates of solute atoms along basal planes are reported to be higher than that along other crystallographic planes according to *ab initio*^29^,^30^. As such, it is reasonable to assume that the Zn/Y atoms tend to segregate into the dislocations along basal planes in the matrix and then diffuse into basal planes of the twin. Then, the supersaturated Zn/Y atoms in the twin spontaneously re-precipitate to form II T-SFs/LPSO, parallel to the basal plane, which are distributing at the original LPSO-TB interface in the deformation twin (Fig. 5d). Two kinds of potential nucleation sites for II T-SFs/LPSO are proposed here. One should be the original misfit dislocations, where a few II T-SFs (~20 nm) were longer than the others owing to the fast diffusion of solute atoms along basal planes in Mg alloy. The other could be LPSO-TB interface, where the residual lattice strain induced by the LPSO-twin interaction should be the driving force for the nucleation of the embryo of LPSO in the twin. The precipitation of II T-SFs/LPSO is beneficial to minimizing the elastic strain owing to the bonding of the solute atom smaller than Mg and the solute atom larger than Mg^31^. This is also accompanied by the migration of the TB, from the dashed lines to solid lines in Fig. 5. Note that the convex border and terraces of the TB illustrated here at the intersection of the twin and LPSO structures, also observed in Figs 3a and 4a, suggesting that the LPSO structure could pin the migration of twin boundaries. Thus this kind of interaction should help to strengthen this Mg alloy to a certain extend.

The misfit dislocations at the LPSO-TB interface, triggering fast diffusion of solute atoms along basal planes in both matrix and twin, are believed to play a significant role during the decomposition of lamellar LPSO structures and precipitation of T-SFs in the twin. We propose the possible mechanisms which are responsible for the microstructure evolution by considering the transmission of solute atoms from the matrix into the deformation twin. One mechanism arises from the dislocation-enhanced diffusion which accelerates the motion Zn/Y atoms from the disordered lamellar LPSO structures of matrix into the deformation twin^32^,^33^,^34^,^35^, and the experiment temperature (573 K) should enhance the diffusion further. Another possible mechanism is shear-driven mechanical mass transport via dislocation motion^6^,^8^,^35^,^36^,^37^. This is presumed to occur only on a short-range scale, i.e. atomic shuffling, and hence, induce deviation from stoichiometry of lamellar LPSO structures at the initial stage of twinning (Fig. 2b). Further, the atomistic simulation revealed that the nucleation and motion of interface disconnections (IDs) along the BP/PB interface would produce a single crystal in pure magnesium during compression or tension^14^. The movement of IDs during deformation may also contribute to the transfer of solute atoms in alloys. From energetics point of view of solute atoms segregating to dislocations at the BP boundary, Kumar *et al*. reported that solutes with a smaller radius than Mg favor the prismatic plane, while solutes with larger radius than Mg favor the basal plane based on DFT calculations^38^. This implies that the Zn atoms (smaller than Mg) and Y atoms (larger than Mg) segregating at misfit dislocations prefer to stay in the deformation twin and in the matrix, respectively. The different stable position of Zn and Y atoms in the BP interface might decompose the possible Zn_6_Y_8_ or Zn_6_Y_8_ (Mg, Zn, Y) clusters in the AB′C′A building blocks^39^, and finally leading to the decomposition of lamellar LPSO or SFs. Significantly, the semi-coherent LPSO-TBs demonstrated here are obviously different from the coherent TB with solute atoms segregations^40^ or TBs cut through by lamellar LPSO structure^28^, since no misfit dislocations exist at the latter cases. Thus the interaction between the interfacial defects and solute atoms strongly influences the stability of secondary phases.

### Role of atomic evolution of lamellar LPSO structures on mechanical properties

The decomposition of LPSO structures followed by re-precipitation SFs in the Mg alloys may also have implications for tailoring the microstructure of alloys to obtain better properties. First, the original lamellar LPSO structures could be considered as strong barriers for the 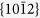 twinning^16^,^19^,^28^, correspondingly improving the strength of Mg alloys. Further, the thin LPSO structures or units of SFs enriched with Zn/Y atoms in the matrix could be twinned (as shown in the twin of Figs 3a and 4a), and in turn the extra energy would be needed to activate the twinning process of lamellar LPSO structures via detwinning of SFs and motion of deformation twin dislocations^28^. Meanwhile, the BP and PBs segregated with Zn/Y atoms (Fig. 4a,c) should impede the propagation of this kind of TB to some extent. It has documented recently that the segregation of solute atoms along grain boundaries or twin boundaries can significantly improve the mechanical properties of Mg alloys via pinning the boundary^40^ and lowering the basal texture^41^,^42^,^43^,^44^. Last but not least, the precipitation of T-SFs could also relax local stress concentration originated from the supersaturating of solute atoms in the twin (as shown in Fig. 3b) and thus retard secondary twinning^45^,^46^ and cracking, then beneficial to the ductility. Therefore, the dynamic evolution of the lamellar LPSO structures in Mg alloys during deformation may contribute to the enhanced mechanical properties.

By means of the atomic resolution scanning transmission electron microscopy, we investigate the redistribution of Zn/Y solute atoms in lamellar LPSO structures caused by twinning in an Mg-Zn-Y alloy. We present a clear compositional signature associated with the evolution of lamellar LPSO structures. The lamellar LPSO structures in the matrix tend to inhibit the propagation of deformation twin, leading to the formation of LPSO-TB interfaces and the resultant misfit dislocations. The diffusion of Zn/Y atoms through the misfit dislocations along unique LPSO-TB interfaces contribute to the redistribution of solute atoms during deformation, resulting in the decomposition of lamellar LPSO structure in matrix and the subsequent re-precipitation of T-SFs/LPSO structures in twins. This study provides new information on the decomposition and re-precipitation of secondary phases during mechanical deformation of engineering alloys, by addressing the influence of atomic scale defects (dislocations here) on the stability of LPSO structures in the Mg-Zn-Y alloy.

## Materials and Methods

### Material fabrications

An Mg_97_Zn_1_Y_2_ (at.%) ingot is prepared using high frequency induction melting in a graphite crucible at approximately 1023 K under an argon atmosphere^16^. Specimens with the dimensions 4 × 4 × 8 mm^3^ were cut from an ingot by electrical discharge machining. The compression direction was parallel to the long axis of the specimens. Hot compression experiments were carried out at 573 K and a strain rate of 1.0 × 10^−1^s^−1^ in a Gleeble-1500 thermal simulation machine. Prior to compression test, the specimens were conductively heated to 573 K at a heating rate of 5 K s^−1^ and held for 180 s for equilibration. After compression, the sample was immediately water quenched to room temperature. The compression failure strain is approximately 36%. The TEM sample extracted from the deformed sample is parallel to the compression axis and near the fractured area.

### TEM characterization

The microstructure analysis of the sample was carried out 8 months after the compression test. The thin foil specimens for microstructure observation were mechanically polished and then ion milled on a cold stage with low angle and low-energy ion beam via Gatan 691 precision ion polishing system. Scanning transmission electron microscopy (STEM) images in this study were acquired on an FEI Titan Cubed 60–300 kV aberration-corrected Scanning TEM (fitted with a high-brightness field emission gun (X-FEG) and double Cs correctors from CEOS, and a monochromator operating at 300 kV). The convergence angle of the electron beam was set to 30 mrad, yielding a probe size of less than 0.10 nm. The collection angle is derived as approximately 63 mrad based on the camera length (CL) is 115 mm, suggesting the collected intensity is dominated by incoherent Z contrast.

## Additional Information

**How to cite this article**: Shao, X. H. *et al*. Deformation twinning induced decomposition of lamellar LPSO structure and its re-precipitation in an Mg-Zn-Y alloy. *Sci. Rep*. **6**, 30096; doi: 10.1038/srep30096 (2016).

## Supplementary Material

Supplementary Information
