# Supplementary material for: Deformation twinning induced decomposition of lamellar LPSO structure and its re-precipitation in an Mg-Zn-Y alloy
Source: Sci Rep. 2016 Jul 20;6:30096. doi: 10.1038/srep30096 (PMC4951811; doi:10.1038/srep30096)
Supplement: Supplementary Information [file srep30096-s1.docx]

**Supplementary Information**

**Deformation twinning induced decomposition of lamellar LPSO structure and its re-precipitation in an Mg-Zn-Y alloy**

X. H. Shao, S. J. Zheng, D. Chen, Q. Q. Jin, Z. Z. Peng & X. L. Ma

*Shenyang National Laboratory for Materials Science, Institute of Metal Research, Chinese Academy of Sciences, 72 Wenhua Road, 110016 Shenyang, China*

Correspondence should be addressed to X. L. Ma (xlma@imr.ac.cn)





Supplementary Fig. S1| The true stress–true strain curve obtained from compression test at 573 K under a constant strain rate of 10^-1^/s.


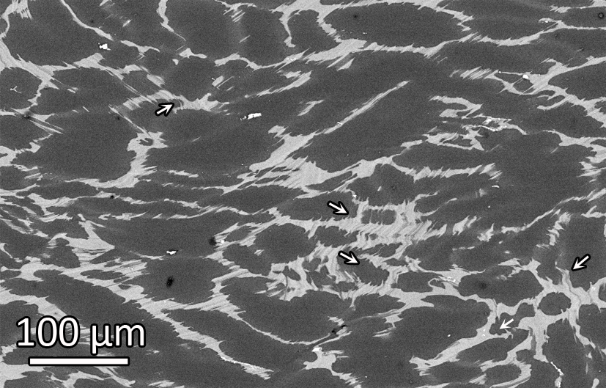


Supplementary Fig. S2| A secondary electron microscopy (SEM) image showing the microstructure of the deformed sample. The deformation kinks observed in LPSO structures were marked by the arrows.


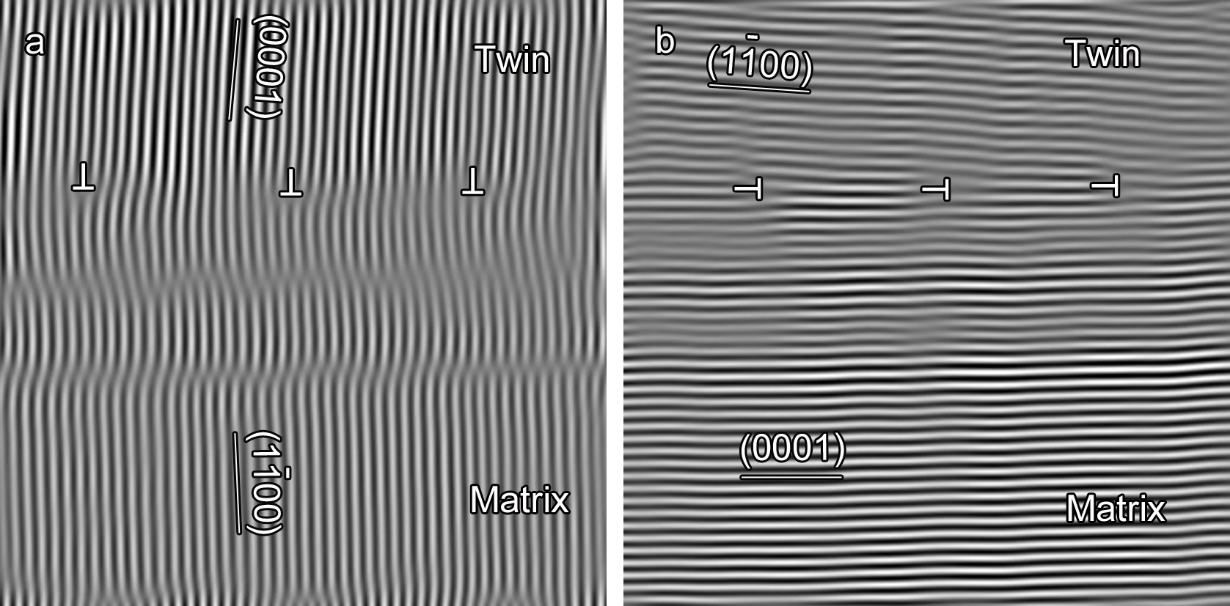


Supplementary Fig. S3 | One-dimensional lattice fringe images obtained using an FFT filtering process showing two kinds of misfit dislocations at the LPSO-TB interface, via (a) (0001) Bragg reflection of twin and {1$\bar{1}$00} Bragg reflection of matrix and (b) (0001) Bragg reflection of matrix and {1$\bar{1}$00} Bragg reflection of twin. The extra half planes demonstrate the distribution of compression strain of the misfit dislocations.


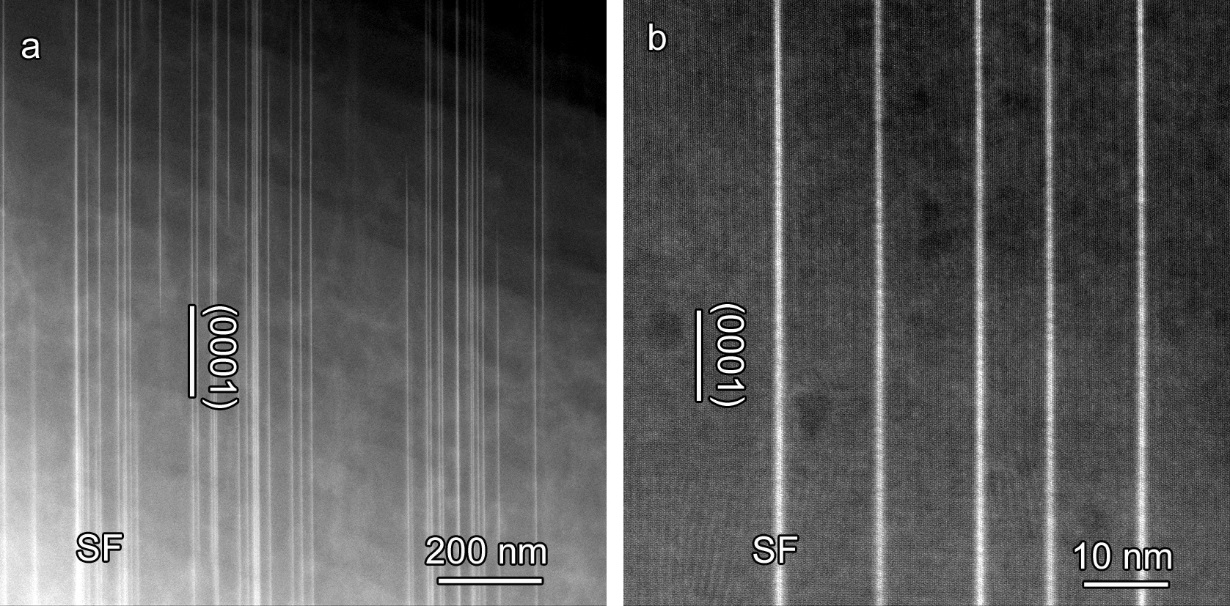


Supplementary Fig. S4 | (a) A low magnification and (b) high magnification STEM images of the undeformed sample which experiences the thermal annealing to 573 K at the heating rate of 5K s^-1^, suggesting the AB´C´A building blocks of the LPSO lamellar and SFs enriched with Zn/Y remain almost the same as at as-cast state and no dislocations at the interface between the Mg layers and SFs/LPSO structures.
